# Assessment of Knowledge, Attitude, and Teaching of the Hall Technique Among Pediatric Dentistry Faculty in Riyadh, Saudi Arabia

**DOI:** 10.3390/dj13060239

**Published:** 2025-05-28

**Authors:** Asma Alshahrani, Haifa Alamro, Fatimah Alanazi, Leqaa Alowaidi, Farah Alhamdan

**Affiliations:** 1 Preventive Dental Sciences Department, College of Dentistry, King Saud bin Abdulaziz University for Health Sciences, P.O. Box 3660, Riyadh 11426, Saudi Arabia; 2 King Abdullah International Medical Research Center, Ministry of National Guard-Health Affairs, P.O. Box 3660, Riyadh 11481, Saudi Arabia; 3 College of Dentistry, King Saud bin Abdulaziz University for Health Sciences, P.O. Box 3660, Riyadh 11426, Saudi Arabia

**Keywords:** Hall Technique (HT), pediatric dentistry, teaching approaches, Saudi Arabia, clinical practice

## Abstract

**Background:** The Hall Technique (HT) in pediatric dentistry is a minimally invasive method for treating carious primary molars by sealing cavities with preformed stainless-steel crowns, eliminating the need for local anesthesia, caries removal or tooth preparation. Objectives: To assess the knowledge, attitude, and teaching of HT among pediatric dentistry faculty in Riyadh, Saudi Arabia. **Methods:** A cross-sectional descriptive study involved 36 pediatric dentistry faculty members from six dental colleges in Riyadh. Data were collected using a validated questionnaire covering demographics and professional information, HT knowledge, attitude, and teaching strategies. **Results:** Thirty-one pediatric dentistry faculty responded to the questionnaire, and 67.74% of participants use HT clinically, primarily for asymptomatic carious primary molars. Most participants learned about HT during postgraduate residency (61.29%), while only 6.45% were introduced to it in undergraduate training. Teaching practices varied, with 51.61% teaching HT to undergraduate and/or postgraduate students, and 16.13% omitting it from their curriculum. Key barriers to HT adoption included limited undergraduate exposure, misconceptions, and a lack of standardized guidelines. Fisher’s Exact Test showed that gender, academic position, and years of experience were significantly associated with HT use. Only years of experience were significantly associated with recommending HT use by undergraduate students. No factors were significantly associated with teaching HT. **Conclusions:** While awareness of HT is high among pediatric dentistry faculty, inconsistent teaching and limited clinical use hinder its full integration. Addressing these barriers through curriculum updates and professional development could enhance the acceptance and quality of HT use in pediatric dentistry in Saudi Arabia.

## 1. Introduction

Pediatric dentistry plays a crucial role in ensuring optimal oral health for children and adolescents. It encompasses a wide range of services, including diagnosis, preventive care, and treatment of dental conditions. Dental caries remains the most prevalent chronic disease among children worldwide, presenting significant challenges due to its high incidence and the potential long-term impact on both oral health and overall well being [1]. Effective caries management is critical for preventing complications, such as pain, infection, and poor nutrition, all of which can affect a child’s growth and development [2]. Traditional caries management often involves the removal of decayed tooth structure, substantial tooth preparation, and the use of local anesthesia. While these techniques are effective, they can be invasive and may cause discomfort or anxiety in young patients, potentially leading to negative dental experiences and the development of dental phobia [3].

Developed by Dr. Norna Hall in the 1990s, the Hall Technique (HT) offers an alternative approach to managing carious primary molars. This method involves sealing lesions with preformed stainless-steel crowns (SSCs) without removing decayed tissue, preparing the tooth, or administering local anesthesia [4]. HT works by halting caries progression through bacterial isolation, depriving bacteria of nutrients, and preserving tooth structure [5]. Aligned with biologically oriented preparation techniques (BOPT), HT is especially beneficial for anxious pediatric patients due to its non-invasive nature [6]. Clinical studies have reported success rates exceeding 90%, with results comparable to or even surpassing conventional methods, while reducing treatment time and costs [7,8,9,10]. The simplicity, effectiveness, and child friendliness of HT increase its acceptance among both patients and parents [11].

A 2020 survey conducted among global pediatric dentists gauged their understanding of the HT. The findings revealed that only 50% (n = 358) of the respondents had comprehensive knowledge of the technique and had used it, while the remaining participants had varying degrees of familiarity with it [12]. In the United States, a study assessing pediatric dentists’ knowledge, attitudes, and factors influencing the use of HT concluded that 96.5% (n = 556) were familiar with the technique, but only 39% (n = 222) implemented it in their practice [13]. Similarly, a study in the United Kingdom found that 96% (n = 89) of specialist pediatric dentists reported using HT in their practice [14].

A study by Al Jundi et al., which assessed the efficacy of pediatric dentistry educational programs in dental schools across the Arabian region, including Jordan, Qatar, and Egypt, found that HT was taught as theoretical instruction in most programs. However, there was a lack of clinical training and implementation of HT in dental clinics [15]. In Saudi Arabia, a study on general practitioners’ knowledge, attitudes, and practices regarding the management of deep carious lesions showed that 55.2% (n = 47) of participants had incorrect definitions of HT, while 80% (n = 96) of pediatric dentists were familiar with it [15]. Additionally, another study in the eastern region of Saudi Arabia, assessing the awareness and use of HT among dental students, interns, and dental professionals, found that 50% of participants were aware of HT, but less than 30% had used it in clinical settings (n = 312) [16].

Several factors contribute to the limited knowledge and usage of HT. These include inadequate training opportunities, such as insufficient exposure to HT in dental schools and professional training, leading to a lack of confidence and skills among practitioners without specialized training [17]. Resistance to change also plays a role, as some clinicians prefer traditional restoration techniques and are reluctant to adopt new methods without solid evidence of their effectiveness and safety [18]. Additionally, misconceptions about the long-term efficacy of HT, such as concerns about pulp necrosis or microleakage, may discourage its use, despite research contradicting these concerns [19].

Limited access to prefabricated stainless-steel crowns and other necessary materials in resource-poor settings can also present a barrier [20].

The results of these studies show varying degrees of knowledge and use of HT among dentists. To our knowledge, in Riyadh, Saudi Arabia, there is a lack of studies in this area. Since HT is a valuable option for treating uncooperative children and is an important technique to be taught and implemented by undergraduate dental students, this research aims to assess the knowledge, attitudes, and teaching of HT among pediatric dentistry faculty in Riyadh, Saudi Arabia.

## 2. Materials and Methods

This cross-sectional descriptive study targeted all pediatric dentistry faculty members from dental colleges in Riyadh, Saudi Arabia, between September and December 2024. The participating institutions included King Saud University (KSU), King Saud bin Abdulaziz University for Health Sciences (KSAU-HS), Princess Nourah Bint Abdulrahman University (PNU), Riyadh Elm University (REU), Dar Al Uloom University (DAU), and Vision College. Faculty members were eligible if they were part of a pediatric dentistry department, held the position of lecturer or higher, and were actively involved in teaching undergraduate and/or postgraduate students.

Institutional Review Board (IRB) approval was obtained from King Abdullah International Medical Research Center (IRB/2648/23). Participation was entirely voluntary, and informed consent was obtained from all respondents before the start of data collection. The study ensured participant anonymity and confidentiality, with no personally identifiable information collected. All data were securely stored and accessed only by the research team. Participants’ email addresses were obtained through an official request sent to their institutions, and no incentives or compensation were offered for participation.

The first phase of data collection involved compiling a list of the current pediatric dentistry faculty members at each dental college. A newly developed questionnaire, based on a thorough review of relevant literature and existing tools used in similar studies, was used for data collection [3,5,14]. The questionnaire was validated by five faculty members using the Aiken index. The questionnaire consisted of three main sections: the first section gathered demographic and professional information, including gender, nationality, academic position, year of graduation from residency, years of experience in the field, and the academic institution they teach at; the second section evaluated the knowledge and usage patterns related to the HT, including sources of knowledge and understanding of its concepts and applications; and the third section focused on the teaching of HT to both undergraduate and postgraduate dental students.

The questionnaire was distributed electronically through email using Google Forms to all pediatric dentistry faculty members (N = 36). Follow-up emails were sent at 2-week intervals, twice, to ensure full participation and comprehensive coverage of the targeted population.

Data analysis was performed using the Statistical Package for the Social Sciences, SPSS 23rd version. Frequency and percentages were used to display categorical variables. Fisher’s exact test was used to test for the presence of association between categorical variables, independent variables, including gender, nationality, academic position, and years of experience were analyzed for their influence on key outcomes (dependent variables,) including current use of HT in practice, teaching HT to various groups, and recommending HT for undergraduate students. The level of significance was set at 0.05.

## 3. Results

### 3.1. Descriptive Statistics

The demographic and professional characteristics of the participants are presented in Table 1. Thirty-one pediatric dentistry faculty members participated in this study, achieving a response rate of approximately 86%. The majority of the participants were females, representing 70.97%. In terms of nationality, Saudi participants dominated with 87.10%. Assistant professors comprised the largest group at 48.39%, associate professors at 25.81%, then professors at 16.13%, while lecturers were the least represented at 9.67%. For graduation year from residency programs, the proportions were balanced among those who graduated before 2010 and between 2010 and 2015, representing 32.26% each, with a slight increase for those who graduated in 2016 or later at 35.48%. Regarding years of experience, the group with 11 to 20 years of experience was the most prevalent at 29.03%, followed by those with more than 20 years at 25.81%, and both the groups with 6 to 10 years and less than 5 years each representing 22.58%. The majority of participants were employed at King Saud University (58.06%), followed by King Saud bin Abdulaziz University for Health Sciences and Princess Nourah Bint Abdul Rahman University, each representing 16.125%.

### 3.2. Knowledge and Attitude

Table 2 shows the knowledge and attitudes of faculty members toward HT. All the participants reported being aware of the Hall Technique. The primary sources of knowledge were postgraduate residency programs at 61.29%, followed by scientific journals, conferences, and workshops at 32.26%. Approximately 67.74% of participants indicated regular use of HT in their clinical practice, with the majority having used it for more than six years (38.1%). The primary indication for HT use was as a treatment option for asymptomatic carious primary molars (52.50%).

### 3.3. Teaching Practices

Teaching practices of HT varied significantly among faculty members. Table 3 outlines the distribution of teaching practices. Among the participants, 83.9% reported teaching HT to undergraduate students and/or postgraduate residents. Among those who taught HT, the predominant methods included didactic lectures (61.5%), hands-on workshops (27%), and clinical demonstrations (11.5%). Finally, when asked about recommending the clinical use of HT by undergraduate students, opinions were nearly split, with 48.4% in favor and 51.1% against. For those who answered “Yes” to using HT by undergraduate students, they believe that the technique introduces students to non-invasive procedures, which adds value to traditional methods. They view HT as a flexible treatment option suitable for various clinical scenarios, especially for children with extensive caries. It is considered a simple, quick, and effective method for managing uncooperative young patients. This experience enhances students’ knowledge of pediatric dentistry, keeps them updated on new treatment options, and strengthens their understanding of traditional techniques, such as conventional crowns. For those who answered “No” to the use of HT by undergraduate students, they argue that the technique might be beyond the scope of general dental practice. They emphasize the importance of mastering traditional methods first, such as tooth preparation for conventional crowns. Concerns were raised about the potential misuse of HT by practitioners with limited experience, as the technique requires careful case selection, which may be challenging for undergraduate students to apply correctly. Additionally, concerns regarding potential impacts on the child’s occlusion were expressed. Some believe HT should only be used in specific cases where traditional methods are not feasible, which demands greater expertise and follow up.

### 3.4. Factors Influencing HT Adoption and Teaching

Table 4 shows the factors associated with the use of HT. Gender was significantly associated with the use of HT (*p* = 0.03), where it was observed that males have a significantly higher rate of use compared to females (100% vs. 54.5%). Years of experience were also significantly associated with the use of HT (*p* = 0.003), where it was observed that the groups with 10 years of experience or less had a significantly higher rate of HT use compared to the groups with more than 10 years of experience. The academic position was also significantly associated with the use of HT (*p* = 0.025), where it was observed that associate professors and professors have a notably lower rate of using HT compared to lecturers and assistant professors. Nationality was not significantly associated with the use of HT.

Table 5 presents the factors associated with teaching HT. Gender, nationality, years of experience, and academic position were all not significantly associated with teaching HT.

Table 6 displays the factors associated with recommending the use of HT by undergraduate students. Years of experience were found to be significantly associated with recommending the use of HT by undergraduate students (*p* = 0.046), where it was observed that the highest rate of recommendation was from those with 5 years of experience or less and those with more than 20 years of experience. Gender, nationality, and academic position were not significantly associated with recommending the use of HT by undergraduate students.

Table 7 demonstrates the association between teaching HT and recommending the use of HT by undergraduate students. No significant association was found between teaching HT and recommending it by undergraduate students.

The distribution of teaching the Hall Technique and the corresponding recommendations for its use by undergraduate students across different academic institutions are illustrated in Table 8. The recommendation rates varied significantly. At King Saud University, 18 respondents teach the technique, with eight recommending its use, leading to a 44.44% recommendation rate. Similarly, at King Saud bin Abdulaziz University for Health Sciences, five respondents teach the technique, but only one recommends its use, with a recommendation rate of 20%. Conversely, at Princess Nourah Bint Abdul Rahman University, out of five respondents teaching the technique, four recommend its use, achieving a high recommendation rate of 80%. At both Riyadh Elm University and the Vision College, one respondent teaches the technique and recommends its use, while at Dar Al Uloom University, the sole respondent teaches but does not recommend the technique.

## 4. Discussion

The Hall Technique (HT) is recognized by all faculty members in pediatric dentistry, reflecting a strong level of awareness among professionals. This aligns with findings from previous studies by Gonzalez et al., Roberts et al., Hussein et al., Dean et al., and Mohamed et al. [12,13,14,21,22]. In contrast, a study by Santamaria et al. [23] showed that the majority of German dentists were unfamiliar with HT, suggesting notable regional variation in awareness and adoption.

In the present study, postgraduate residency programs were the primary source of HT knowledge, consistent with Crystal et al., who reported that 65% of pediatric program directors used HT clinically and 90% included it in their teaching [17]. Clinically, HT was primarily used for asymptomatic carious primary molars, with 67.74% of participating faculty reporting active use. This is comparable to the findings of Roberts et al., who reported a 96% usage rate among UK pediatric dentists [14]. However, global uptake remains inconsistent, as reflected in the lower usage rates reported by Hussein et al. and Mohamed et al. in Qatar [12,22].

The present study also revealed considerable variability in the inclusion of HT in undergraduate dental curricula. While 51.6% of faculty reported teaching HT at the undergraduate level, 16.13% excluded it entirely, highlighting inconsistent educational practices. These findings are somewhat aligned with Gonzalez et al., who found only 17% support for undergraduate HT teaching [13], but contrast with Roberts et al. and Hussein et al., who reported higher endorsement rates of 85% and 28%, respectively [12,14].

Encouragingly, Roberts et al. noted that 90% of UK pediatric specialists support teaching HT at the undergraduate level, and the technique is now included in the curricula of all UK and New Zealand dental schools, as well as several European institutions [14]. Likewise, 60% of Malaysian pediatric dentists favored undergraduate HT instruction [24]. This international support is grounded in robust clinical evidence; randomized controlled trials and systematic reviews have consistently demonstrated the clinical superiority of HT-preformed metal crowns over conventional restorations for multi-surface lesions, with benefits including improved longevity, cost effectiveness, and patient acceptance [14,24,25].

Despite some concerns regarding aesthetics and long-term outcomes, the majority of faculty in this study expressed a positive attitude toward HT, recognizing its clinical advantages. Notably, years of experience and academic position were significantly associated with HT use. Faculty with 10 years or less of experience were more likely to use HT, and lecturers and assistant professors had higher usage rates than associate professors and professors. These findings may reflect generational differences in training and openness to minimally invasive techniques. Interestingly, nationality was not significantly associated with HT use, suggesting that professional role and clinical experience are more influential than cultural background.

Institutional differences in teaching practices were also observed. For example, Princess Nourah University had an 80% recommendation rate for HT instruction at the undergraduate level, whereas King Saud bin Abdulaziz University reported only 20%. Such discrepancies may be influenced by faculty training backgrounds, institutional guidelines, or administrative priorities.

This study is not without limitations. The most significant limitation is the limited generalizability due to the small sample size and the concentration of participants from a single institution (King Saud University, 58.06%). Additionally, nearly half of the participants were assistant professors, limiting perspectives from more senior faculty. The reliance on self-reported data introduces potential for recall bias and social desirability bias, which may have inflated reported knowledge and attitudes. Future research should aim to include a more diverse and representative sample from multiple regions and institutions.

From an educational standpoint, the findings reveal significant variation in how HT is taught. The study did not evaluate the effectiveness of instructional methods, such as lectures versus hands-on workshops, nor did it include student perspectives. Gathering feedback from students on their confidence in using HT could provide valuable insights into the teaching process. Furthermore, while years of experience appeared to influence HT recommendations, this association did not reach statistical significance. Understanding where and how faculty learned HT—whether during formal education or clinical experience—may help inform targeted faculty development initiatives.

To strengthen HT adoption and instruction, several strategies are recommended. Integrating HT into undergraduate curricula will ensure early exposure and competence among future practitioners. Faculty development through workshops and continuing education is critical to standardize teaching and clinical application. Institutional policies should include HT in clinical guidelines and allocate resources, such as stainless steel crowns. Finally, mentorship from experienced faculty can foster broader acceptance and confidence in HT use.

## 5. Conclusions

This study reveals a high level of awareness and acceptance of the Hall Technique among pediatric dentistry faculty in Riyadh, Saudi Arabia. However, inconsistencies in teaching practices and limitations in clinical use suggest barriers to broader adoption. Addressing these challenges through curriculum reform, expanded training opportunities, and standardized institutional policies will be key to integrating HT more effectively into both education and practice. Promoting a supportive learning environment and dispelling misconceptions can facilitate the technique’s adoption, ultimately enhancing the quality of pediatric dental care in the region.

## Figures and Tables

**Table 1:** Demographic and professional characteristics of pediatric dentistry faculty.

Variable	Frequency	Percentage (%)
**Gender**		
Female	22	70.97
Male	9	29.03
**Nationality**		
Saudi	27	87.10
Non-Saudi	4	12.90
**Academic position**		
Lecturer	3	9.67
Assistant Professor	15	48.39
Associate Professor	8	25.81
Professor	5	16.13
**Year of graduation from residency**		
Before 2010	10	32.26
2010–2015	10	32.26
2016 or later	11	35.48
**Years of experience in pediatric dentistry**		
Less than 5 years	7	22.58
6 to 10 years	7	22.58
11 to 20 years	9	29.03
More than 20 years	8	25.81
**The academic institution at which you are teaching:**		
King Saud University	18	58.06
King Saud bin Abdulaziz University for Health Sciences	5	16.125
Princess Nourah Bint Abdul Rahman University	5	16.125
Riyadh Elm University	1	3.23
Dar Al Uloom University	1	3.23
Vision College	1	3.23

**Table 2:** Knowledge and attitude towards HT.

Variable	Frequency	Percentage (%)
**Are you familiar with the HT?**		
Yes	31	100%
No	0	0
**Where did you learn about the Hall Technique (HT)?**		
Postgraduate residency programs	19	61.29
Scientific journal/online forum/conferences/workshops	10	32.26
Undergraduate dental colleges	2	6.45
**Do you currently use the HT in your practice?**		
Yes	21	67.74
No	10	32.26
**How long have you been using HT?**		
1 to 2 years	2	9.52
3 to 4 years	7	33.33
5 to 6 years	4	19.05
More than 6 years	8	38.1
**In what cases do you use HT?**		
Treatment of choice for asymptomatic carious primary molars	2	5
Treatment option for asymptomatic carious primary molars	21	52.50
Only when unable to use conventional crowns in carious primary molars	14	35
Never	3	7.50

**Table 3:** Teaching HT by pediatric dentistry faculty members.

Variable	Frequency	Percentage (%)
**Are you currently teaching HT to any of the following?**		
Undergraduate dental students	6	19.35
Postgraduate residents	10	32.26
Both undergraduate and postgraduate	10	32.26
Undergraduate and/or postgraduate	26	83.9
Do not teach HT	5	16.13
**Method of Teaching**		
Didactic lectures	16	61.5
hands-on workshops	7	27
Clinical demonstrations	3	11.5
**Do you recommend using HT by undergraduate students?**		
Yes	15	48.4
No	16	51.6

**Table 4:** Factors associated with the use of HT by pediatric dentistry faculty.

Variable	Do You Currently Use HT in Your Practice?		*p*-Value
	No	Yes	
Gender			0.03 *
Male	0 (0%)	9 (100%)	
Female	10 (45.5%)	12 (54.5%)	
Nationality			0.277
Saudi	10 (37%)	17 (63%)	
Non-Saudi	0 (0%)	4 (100%)	
Years of experience			0.003 *
5 years and less	0 (0%)	7 (100%)	
6 to 10 years	0 (0%)	7 (100%)	
11–20 years	6 (66.7%)	3 (100%)	
More than 20 years	4 (50%)	4 (50%)	
Academic position			0.025 *
Lecturer	0 (0%)	3 (100%)	
Assistant Professor	2 (13.3%)	13 (86.7%)	
Associate Professor	5 (62.5%)	3 (37.5%)	
Professor	3 (60%)	2 (40%)	

* Significant at level 0.05.

**Table 5:** Factors associated with the HT teaching by pediatric dentistry faculty.

Variable	Are You Currently Teaching HT?		*p*-Value
	No	Yes	
Gender			1.000
Male	1 (11.1%)	8 (88.9%)	
Female	4 (18.2%)	18 (81.8%)	
Nationality			1.000
Saudi	5 (18.5%)	22 (81.5%)	
Non-Saudi	0 (0%)	4 (100%)	
Years of experience			0.289
5 years and less	0 (0%)	7 (100%)	
6 to 10 years	1 (14.3%)	6 (85.7%)	
11–20 years	1 (11.1%)	8 (88.9%)	
More than 20 years	3 (37.5%)	5 (62.5%)	
Academic position			0.477
Lecturer	0 (0%)	3 (100%)	
Assistant Professor	2 (13.3%)	13 (86.7%)	
Associate Professor	1 (12.5%)	7 (87.5%)	
Professor	2 (40%)	3 (60%)	

* Significant at level 0.05.

**Table 6:** Factors associated with recommending using HT by undergraduate students.

Variable	Do You Recommend Using HT by Undergraduate Students?		*p*-Value
	No	Yes	
Gender			0.252
Male	3 (33.3%)	6 (66.7%)	
Female	13 (59.1%)	9 (40.9%)	
Nationality			0.333
Saudi	15 (55.6%)	12 (44.4%)	
Non-Saudi	1 (25%)	3 (75%)	
Years of experience			0.046 *
5 years and less	1 (14.3%)	6 (85.7%)	
6 to 10 years	5 (71.4%)	2 (28.6%)	
11–20 years	7 (77.8%)	2 (22.2%)	
More than 20 years	3 (37.5%)	5 (62.5%)	
Academic position			0.686
Lecturer	2 (66.7%)	1 (33.3%)	
Assistant Professor	6 (40%)	9 (60%)	
Associate Professor	5 (62.5%)	3 (37.5%)	
Professor	3 (60%)	2 (40%)	

* Significant at level 0.05.

**Table 7:** Association between teaching HT and recommending using HT by undergraduate students.

Variable	Do You Recommend Using HT to Undergraduate Students?		*p*-Value
	No	Yes	
Are you currently teaching HT to any of the following groups?			0.333
No	4 (80%)	1 (20%)	
Yes	12 (46.2%)	14 (53.8%)	

* Significant at level 0.05.

**Table 8:** Teaching and using HT by undergraduate students by academic institution.

Academic Institution	Total Respondents	Teaching Groups	Recommending the Use of HT by Undergraduate Students No. (%)
King Saud University	18	Postgraduate residents (6)	8 (44.44)
		Both undergraduate and postgraduate (8)	
		do not teach HT (4)	
King Saud bin Abdulaziz University for Health Sciences	5	Postgraduate residents (3)	1 (20)
		Undergraduate dental students (2)	
Princess Nourah Bint Abdul Rahman University	5	Both undergraduate and postgraduate (1)	4 (80)
		Undergraduate dental students (3)	
		Do not teach HT (1)	
Riyadh Elm University	1	Both undergraduate and postgraduate	1 (100)
Dar Al Uloom University	1	Postgraduate residents	0
Vision College	1	Undergraduate dental students	1 (100)

## Data Availability

The original contributions presented in this study are included in the article.
